# Perioperative chemotherapy for locally advanced gastric cancer in Japan: current and future perspectives

**DOI:** 10.1007/s00595-019-01896-5

**Published:** 2019-10-14

**Authors:** Masanori Tokunaga, Yuya Sato, Masatoshi Nakagawa, Tomoki Aburatani, Takatoshi Matsuyama, Yasuaki Nakajima, Yusuke Kinugasa

**Affiliations:** Department of Gastrointestinal Surgery, Tokyo Medical and Dental University, 1-5-45 Yushima, Bunkyo-ku, Tokyo, Japan

**Keywords:** Gastric cancer, Adjuvant chemotherapy, Neoadjuvant chemotherapy, Perioperative chemotherapy, D2 gastrectomy

## Abstract

The standard treatment for locally advanced gastric cancer differs across the world. In western countries, perioperative chemotherapy or postoperative adjuvant chemoradiotherapy are the preferred treatment options, whereas in Asia, D2 gastrectomy followed by postoperative adjuvant chemotherapy is standard. In Japan, adjuvant chemotherapy with S-1 is the standard treatment for pStage II gastric cancer, whereas adjuvant chemotherapy with a doublet regimen is preferred for pStage III gastric cancer. The efficacy of preoperative neoadjuvant chemotherapy using S-1 plus cisplatin, has been investigated in selected patients with expected poor survival outcomes. To expand the indications for neoadjuvant chemotherapy, a clinical trial investigating the efficacy of preoperative S-1 plus oxaliplatin in patients with cStage III (cT3-4N1-3) gastric cancer (JCOG1509) is ongoing in Japan. The addition of immune checkpoint inhibitors to cytotoxic chemotherapy also seems promising and is being investigated in international randomized clinical trials. Although we have to await the final results of these studies, preoperative neoadjuvant chemotherapy is a promising treatment strategy and likely to become standard treatment for locally advanced gastric cancer in Japan.

## Introduction

Gastric cancer is the fifth most common type of cancer and the third leading cause of cancer-related deaths worldwide [1]. The incidence of gastric cancer differs among regions, with the highest incidences reported in East Asian countries, including Japan, Korea, and China. Similarly, patient clinicopathological characteristics differ and early-stage gastric cancer is more frequently observed in East Asia.

The principle of treatment for gastric cancer is the same, regardless of region, with curative resection the mainstay option, although different preoperative and postoperative adjuvant treatments have developed in different regions. In Europe, perioperative chemotherapy is standard for locally advanced gastric cancer. The efficacy of this approach was confirmed by the MAGIC trial, which showed better survival of patients who received perioperative chemotherapy using epirubicin, cisplatin, and infused fluorouracil than of those treated without perioperative chemotherapy (HR, 0.75; 95% CI, 0.60–0.93) [2]. This triplet regimen was standard before the superiority of perioperative fluorouracil plus leucovorin, oxaliplatin, and docetaxel (FLOT) was demonstrated in the FLOT4 study [3], wherein the hazard ratio for overall survival achieved in the FLOT arm versus the standard treatment arm was 0.77 (95% CI, 0.63–0.94).

In America, postoperative adjuvant chemoradiotherapy is preferred as the standard treatment, based on the efficacy demonstrated in the INT0116 study, in which the hazard ratio for death in the chemoradiotherapy group versus the surgery-only group was 0.74 (95% CI, 0.60–0.92) [4]. Conversely, in East Asia, gastrectomy with postoperative adjuvant chemotherapy is preferred, based on the results of clinical trials, although preoperative neoadjuvant chemotherapy is given to selected patients [5–8].

Accordingly, patients with gastric cancer receive different perioperative treatments depending on the country where they are treated; thus, the future perspectives for patients also differ among countries. In this article, we review the current standard treatments for locally advanced gastric cancer and future perspectives in Japan.

## Standard surgery for locally advanced gastric cancer in Japan

Curative resection is essential to achieve a good prognosis for patients with locally advanced gastric cancer, and therefore, it should be the mainstay of treatment. Gastrectomy with D2 lymph node dissection (D2 gastrectomy) is the standard surgical resection procedure in Japan and Europe [9, 10]. In the Japanese Gastric Cancer Treatment Guidelines (JGCA guidelines), standard gastrectomy is defined as “the principal surgical procedure performed with curative intent. It involves resection of at least two-thirds of the stomach with a D2 lymph node dissection” [10]. The efficacy of more extended and aggressive procedures has been evaluated by the Japan Clinical Oncology Group (JCOG, Table 1); however, none has demonstrated superior benefit, including prophylactic para-aortic lymph node dissection (JCOG9501), the left thoracoabdominal approach (JCOG9502), or omentobursectomy (JCOG1001) [11–13]. The JCOG0110 trial also ruled out the superiority of splenectomy and confirmed the non-inferiority of spleen-preserving gastrectomy for patients with upper third advanced gastric cancer without greater curvature line involvement [14]. These results imply that more extensive operations would not improve long-term outcomes and might even cause postoperative complications and potentially worsen long-term survival outcomes [13–16]. Therefore, incorporating perioperative chemotherapy into the standard D2 gastrectomy protocol, rather than performing extended operations, seems to be a better approach for improving long-term outcomes.

**Table 1:** Randomized studies that investigated the efficacy of extended surgery

Trial	Phase	Design	Patients	Arms	No. of patients	Hazard ratio for OS (95% CI)	Results
JCOG9501 [11]	III	Superiority	AGC (≥ cT3)	S: D2	263		Negative
				E: D2 + PAND	259	1.03 (0.77–1.37)	
JCOG9502 [12]	III	Superiority	AGC with ≤ 30 mm esophageal invasion	S: TH approach	82		Negative**
				E: LTA approach	85	1.36 ( 0.89–2.08)	
JCOG0110 [14]	III	Non-inferiority	Upper third AGC	S: splenectomy	254		Positive
				E: spleen preservation	251	0.88 (0.67–1.16)*	
JCOG1001 [13]	III	Superiority	AGC (≥ cT3)	S: omentectomy	602		Negative***
				E: omentobursectomy	602	1.05 (0.81–1.37)	

*AGC* advanced gastric cancer, *S* standard arm, *E* experimentary arm, *PAND* para-aortic lymph node dissection, *TH* transhiatal, *LTE *left thoracoabdominal, *CI* confidential interval

*90.7% CI; **terminated at interim analysis before completion of patient accrual; ***terminated at interim analysis after completion of patient accrual

D2 gastrectomy can be performed by laparoscopy, with technical feasibility comparable to open gastrectomy already confirmed by randomized controlled trials conducted in East Asia [17–19]. In Japan, a multicenter randomized controlled trial aimed at demonstrating the non-inferiority of laparoscopic D2 gastrectomy versus open D2 gastrectomy in patients with cT2-4N0-2 gastric cancer is ongoing (JLSSG [Japanese Laparoscopic Surgery Study Group] 0901), with patient accrual already completed and the final analysis expected in 2021. Furthermore, a Chinese series has already demonstrated the non-inferiority of laparoscopic D2 gastrectomy, and this procedure should expand rapidly in the near future [20]. Potentially, robot-assisted D2 gastrectomy is also technically feasible, although long-term outcomes are yet to be evaluated in prospective studies in Japan [21].

## Standard Postoperative adjuvant chemotherapy in Japan (Table 2)

**Table 2:** Trials investigating the efficacy of postoperative adjuvant chemotherapy following D2 gastrectomy

Trial/authors	Phase	Patients	Arms	No. of patients	5Y-RFS (%)	5Y-OS (%)	Hazard ratio for OS (95% CI)
ACTS-GC [5, 6]	III	pStage II/III	S: surgery alone	530	53.1	61.1	
			E: surgery + S-1	529	65.4	71.7	0.66 (0.54–0.82)
NSAS-GC [22]	III	pT2N1-2	S: surgery alone	95		73	
			E: surgery + UFT	93		86	0.48 (0.26–0.89)
CLASSIC [7]	III	pStage II/III	S: surgery alone	515	68	78	
			E: surgery + CapOx	520	53	69	0.66 (0.51–0.85)
JACCRO GC-07 [28]	III	pStage III	S: surgery alone	459	50*		
			E: surgery + DS	454	66*	–	0.63 (0.40–0.99)***
Takahari et al. [23, 24]	II	pStage III	Surgery + SP	63	74*	85**	
							–
J-CLASSIC [25, 27]	II	pStage II/III	Surgery + CapOX	100	pStage II, 88*	pStage II, 93**	
					pStage III,68*	pStage III, 79**	–
SOXaGC [26, 27]	II	pStage III	Surgery + SOX	62	71*	76**	
							–

*AGC* advanced gastric cancer, *S* standard arm, *E* experimentary arm, *CapOX* capecitabine + oxaliplatin, *DS* docetaxel + S-1, *SP* S-1 + cisplatin, *SOX* S-1 + oxaliplatin

*3y-RFS; **3Y-OS; ***99.99% CI

In Japan, postoperative adjuvant chemotherapy with S-1 for 1 year became a standard treatment after the ACTS-GC trial (the adjuvant chemotherapy trial of TS-1 for gastric cancer) demonstrated its superiority over surgery alone [5]. In that trial, 1059 patients with pStage II/III gastric cancer, according to the thirteenth edition of the Japanese classification of gastric carcinoma (JCGC), were allocated randomly to either a surgery-alone arm or surgery with S-1 arm, between 2001 and 2004. The study was terminated at the first interim analysis, because the significance level of differences in survival was close to the predetermined threshold for the interim analysis. Improved 5-year overall survival of the surgery with S-1 group was confirmed by the 5-year follow-up results (71.7% vs. 61.1%; HR, 0.669; 95% CI, 0.540–0.828) [6].

Another clinical trial showing the superiority of surgery with uracil-tegafur (UFT) over surgery alone for patients with pT2N1-2 was conducted before the ACTS-GC trial. The planned sample size was 488 patients, but patient accrual was slower than expected and the recruitment of patients was terminated midway through the trial. In total, 190 patients were randomized and the final analysis showed better 5-year survival rates in the surgery with UFT arm (86% vs. 73%; HR, 0.48; 95% CI, 0.26–0.89) [22].

In subsequent subset analyses of the ACTS-GC trial, 5-year survival rates of the surgery with S-1 group of patients with pStage II, IIIA, and IIIB disease were 84.2%, 67.1%, and 50.2%, respectively, with hazard ratios of 0.509 (95% CI, 0.338–0.765), 0.708 (95% CI, 0.510–0.983), and 0.791 (95% CI, 0.520–1.205), respectively, indicating that more intensified perioperative chemotherapy would be necessary to improve the overall dismal survival outcomes of patients with pStage III disease. Accordingly, several phase II trials investigating the efficacy of similar doublet regimens were conducted. Takahari et al. [23, 24] evaluated postoperative S-1 plus cisplatin for patients with pathological stage III gastric cancer. The regimen was tolerable with slight modification and provided better 3-year recurrence-free and overall survival rates (74.1% and 84.5%, respectively). The feasibility of capecitabine plus oxaliplatin (CapOX), which was the same regimen used in the capecitabine and oxaliplatin adjuvant study in stomach cancer (CLASSIC) study conducted in south Korea, and S-1 plus oxaliplatin (SOX), were also evaluated in phase II trials [25, 26]. These regimens seemed feasible for Japanese patients with gastric cancer following D2 gastrectomy. The survival outcomes of patients treated with CapOX or SOX were also evaluated, showing 3-year recurrence-free and overall survival rates of 67.8–70.9% and 75.7–79.3%, respectively [27].

JACCRO (Japan Clinical Cancer Research Organization) GC-07 was a randomized controlled trial that evaluated the superiority of postoperative S-1 plus docetaxel (DS) over S-1 monotherapy in patients with pStage III gastric cancer. The study was terminated at the second interim analysis, because far better RFS of the S-1 plus docetaxel group was demonstrated (HR, 0.632; 99.99% CI, 0.400–0.998) with 3-year RFS rates of 66% in the S-1 plus docetaxel group and 50% in the S-1 monotherapy group [28].

For pStage II gastric cancer, the ACTS-GC trial revealed satisfactory survival outcomes (84.2% of 5-year OS); thus, the possible feasibility of shorter S-1 duration was evaluated in JCOG1104. The study was terminated at the first interim analysis with HR for the 6-month group versus the 12-month group being 2.52 (95% CI, 1.11–5.77), which met the prespecified criteria for early termination. The 3-year RFS of the 6-month and 12-month groups were 89.8% and 93.1%, respectively. D2 gastrectomy followed by S-1 for 1 year thus remains the standard treatment for pStage II gastric cancer [29].

## Standard Preoperative neoadjuvant chemotherapy in Japan (Table 3)

**Table 3:** Trials investigating the efficacy of preoperative neoadjuvant chemotherapy in Japan (including ongoing and international trials)

Trial/authors	Phase	Patients	Arms	No. of patients	5Y-RFS	5Y-OS	Hazard ratio for OS (95% CI)
JCOG0405 [8]	II	Extended LN metastasis	SP + surgery	51		53	
JCOG1002 [32, 33]	II	Extended LN metastasis	DCS + surgery + S-1	52		55%	
JCOG0501 [30, 31]	III	Type 4/Large type 3	S: surgery + S-1	149	48%**	62%***	
			E: SP + surgery + S-1	151	48%**	61%***	0.92 (0.68–1.23)
JCOG1509 [35]	III	cStrage III	S: surgery + S-1/DS	235*			
			E: SOX + surgery + S-1/DS	235*	Ongoing		
JCOG1301C [37]	rPII	Extended LN metastasis	S: surgery + S-1	65*			
			E: SP/T-mab + surgery + S-1	65*	Ongoing		
KEYNOTE-585 [39]	III	T3-4Nany/TanyN +	S: surgery + peri-operative XP/FP/FLOT and placebo	430*			
			E: surgery + peri-operative XP/FP/FLOT and pempronizumab	430*	Ongoing		

*LN* lymph node, *SP* S-1 + cisplatin, *DCS* docetaxel + cisplatin + S-1, *SOX* S-1 + oxaliplatin, *T-mab* trastuzumab, *XP* capecitabine + cisplatin, *FP* 5-FU + cisplatin, *FLOT* 5-FU + leucovorin + oxaliplatin + docetaxel

*Planned number of patients recruited for the study; **3Y-PFS; ***3Y-OS

In Japan, the standard treatment for locally advanced gastric cancer is surgery with postoperative adjuvant chemotherapy; however, the efficacy of preoperative neoadjuvant chemotherapy has also been investigated for selected patients with expected poor survival outcomes, such as those with scirrhous type (macroscopic type 4) gastric cancer or gastric cancer with extensive lymph node metastasis. The JCOG0501 is a multicenter randomized controlled trial aimed at demonstrating the efficacy of preoperative neoadjuvant chemotherapy with S-1 plus cisplatin for patients with type 4 or large type 3 (≥ 8 cm in maximum diameter) gastric cancer [30]. Unexpectedly, survival outcomes were similar in the two groups, with 3-year RFS rates of 60.9% and 62.4% for patients who received preoperative neoadjuvant chemotherapy versus those who did not, respectively (hazard ratio, 0.916; 95% CI, 0.679–1.236) [31].

In gastric cancer patients with extensive nodal metastasis, defined as bulky (≥ 30 mm in diameter) supra-pancreatic lymph nodes or enlarged (≥ 10 mm in diameter) para-aortic lymph nodes, the efficacy of preoperative neoadjuvant chemotherapy has been investigated in phase II studies. The JCOG0405, which evaluated the efficacy of preoperative neoadjuvant chemotherapy with 2–3 courses of S-1 plus cisplatin, demonstrated far better 3- and 5-year overall survival rates than expected (59% and 53%, respectively) [8].

The JCOG1002 investigated the efficacy of a triplet regimen with docetaxel, cisplation, and S-1, but the primary endpoint of the clinical response rate of 57.7% was less than the threshold value of 65% [32]. Moreover, the 5-year overall survival rate (54.9%) was similar to and did not exceed that of the JCOG0405. Accordingly, preoperative neoadjuvant chemotherapy with S-1 plus cisplatin remains a standard treatment for patients with extensive nodal metastasis [8, 33].

## Pre- and postoperative adjuvant chemotherapy recommended in the JGCA guidelines

The English version of the Japanese gastric cancer treatment guidelines (ver. 4) recommends S-1 monotherapy as postoperative adjuvant chemotherapy for patients with pStage II/III gastric cancer. On the other hand, the latest Japanese version of the Japanese gastric cancer treatment guidelines (ver. 5), which is not yet available in English, recommends CapeOX as well as S-1 as evidence level A regimens. SOX is also recommended in this guideline as an evidence level B regimen.

Recommendations for preoperative neoadjuvant chemotherapy are limited, although these guidelines state that preoperative neoadjuvant chemotherapy with S-1 plus cisplatin followed by gastrectomy with para-aortic lymph node dissection can provide satisfactory survival outcomes for patients with extensive lymph node metastasis including para-aortic lymph nodes. Therefore, this treatment strategy could be recommended for institutions with sufficient expertise in para-aortic lymph node dissection.

## Ongoing randomized controlled trials

Preoperative neoadjuvant chemotherapy is a promising treatment strategy aimed at improving the dismal outcomes of patients with pStage III gastric cancer. However, clinical stage does not necessarily reflect pathological stage, and contamination of pStage I among candidates for neoadjuvant chemotherapy is a problem, particularly in Japan, where gastric cancer is diagnosed at an early stage in more than half of the patients.

A prospective cross-sectional study, the JCOG1302A, was conducted to prospectively evaluate the correlations between clinical and pathological stage [34]. A total of 1250 patients with cT2-4a locally advanced gastric cancer were enrolled, and cT and cN, and cStage were compared with pT and pN, and pStage. In this study, cT was estimated based on computed tomography and esophagogastroduodenoscopy findings, and lymph nodes larger than 10 mm in long-axis diameter or 8 mm in short-axis diameter were defined as positive. Based on the results of the JCOG1302A, patients with cStage III according to the seventh edition of the TNM classification (cT3-4N1-3) were judged as suitable candidates for preoperative neoadjuvant chemotherapy. The contamination rate of pStage I was 6.5% and the sensitivity for pStage III was 64.5%.

A multicenter randomized controlled trial, the JCOG1509, was subsequently commenced to confirm the efficacy of preoperative neoadjuvant chemotherapy with S-1 plus oxaliplatin in patients with cStage III gastric cancer [35]. The inclusion criteria for the study were decided based on the results of the JCOG1302A, and 470 patients are expected to be enrolled by March 2022.

For patients with HER2-positive gastric cancer, the addition of trastuzumab to perioperative cytotoxic chemotherapy is a plausible treatment option to improve survival outcomes [36]. This is under investigation in a randomized phase II study (JCOG1301C) to evaluate the superiority of preoperative neoadjuvant S-1, cisplatin, plus trastuzumab over S-1 plus cisplatin [37]. A total of 130 patients will be recruited by March, 2021. The addition of immune checkpoint inhibitors to preoperative neoadjuvant chemotherapy or postoperative adjuvant chemotherapy is another promising treatment strategy and international trials are currently underway [38, 39].

## Standard treatment in other East Asian countries

The incidence of gastric cancer in other East Asian countries is similar to that in Japan. In these countries, D2 gastrectomy is standard and minimally invasive surgery is also being performing increasingly. In Korea, postoperative adjuvant chemotherapy with capecitabine plus oxaliplatin became a standard treatment for locally advanced gastric cancer after the CLASSIC trial [7], which showed a 3-year disease-free survival of 74% in the surgery followed by chemotherapy group and 59% in the surgery alone group, with a hazard ratio of 0.56 (95% CI, 0.44–0.72) [40].

The efficacy of adjuvant chemoradiation following D2 gastrectomy was also investigated in the ARTIST trial, in which 458 patients undergoing curative resection were allocated to either an adjuvant chemotherapy group (*n* = 228) or an adjuvant chemoradiotherapy group (*n* = 230). Although survival outcome was slightly better in the chemoradiotherapy group than in the chemotherapy alone group, the difference was not significant (78.2% vs. 74.2%, *p* = 0.0862). However, subset analysis indicated the potential benefit of adjuvant chemoradiotherapy in node-positive locally advanced gastric cancer [41], and the ARTIST 2 trial was conducted to investigate the superiority of S-1 plus oxaliplatin or S-1 plus oxaliplatin with chemoradiotherapy to S-1 monotherapy in patients with node-positive stage II/III gastric cancer. The results showed the superiority of S-1 plus oxaliplatin (HR 0.617; *p* = 0.016) and S-1 plus oxaliplatin with chemoradiotherapy (HR 0.686; *p* = 0.057), but did not show an additional effect of radiotherapy on the doublet regimen [42].

The efficacy of pre-operative neoadjuvant chemotherapy with docetaxel, oxaliplatin, and S-1 is under investigation in the PRODIGY trial (NCT01515748) in Korea. Patient recruitment has been completed and final results are imminent.

## Discussion

Perioperative adjuvant chemotherapy or postoperative adjuvant chemoradiation is standard treatment for locally advanced gastric cancer in western countries [2–4]. In contrast, gastrectomy with postoperative adjuvant chemotherapy is the standard treatment in East Asia [5–7]. Nonetheless, the superiority of perioperative chemotherapy is still being explored and may become standard in the future.

The ACTS-GC trial demonstrated the efficacy of postoperative adjuvant chemotherapy with S-1 for patients with pStage II/III gastric cancer, but found unsatisfactory 5-year survival rates for those with pStage IIIA and IIIB, of 67.1% and 50.2%, respectively, according to the thirteenth edition of the Japanese classification of gastric carcinoma, highlighting the need for improvement [5, 6]. Accordingly, the efficacy of intensified postoperative adjuvant chemotherapy with a doublet regimen was investigated and the JACCRO GC-07 confirmed the superiority of S-1 plus docetaxel over S-1 monotherapy [28]. Consequently, postoperative adjuvant chemotherapy with doublet regimens became standard treatments for pStage III gastric cancer.

After gastrectomy, and particularly total gastrectomy, patients frequently suffer from post-gastrectomy syndromes, such as weight loss, dumping syndrome, or anemia; all of which can result in delayed commencement or discontinuation of postoperative adjuvant chemotherapy. Moreover, postoperative complications, with an incidence range of 15–50%, also may affect the dose intensity of postoperative adjuvant chemotherapy [13, 15, 43, 44]. Accordingly, patients with pStage II/III do not necessarily receive adjuvant chemotherapy with expected dose intensity. On the other hand, preoperative neoadjuvant chemotherapy is not affected by post-gastrectomy syndromes or postoperative complications. Therefore, these patients can benefit from receiving more intensified chemotherapy before, rather than after gastrectomy. Other potential advantages of neoadjuvant chemotherapy are an increased R0 resection rate and earlier commencement of systemic chemotherapy, both of which may contribute to improved long-term survival outcomes [45]. The efficacy of preoperative neoadjuvant chemotherapy was investigated in selected patients with expected poor survival outcomes and subsequently became the standard treatment for advanced gastric cancer with extended lymph node metastasis [8]. To expand the indications of neoadjuvant chemotherapy, several clinical trials are ongoing in Japan [35, 37] and other Asian countries.

Japanese surgeons/oncologists should take recent rapid globalization into account and cooperate with their western counterparts when developing new treatment strategies. While standard perioperative chemotherapy differs between west and east, clinical experience needs to be shared to establish more promising treatment strategies overall for locally advanced gastric cancer.

In conclusion, the current standard treatment for locally advanced gastric cancer in Japan is D2 gastrectomy followed by adjuvant chemotherapy, except for some specific types of gastric cancer. The efficacy of preoperative neoadjuvant chemotherapy is under investigation, and expected to become a standard treatment for locally advanced gastric cancer in Japan.
