# Correction to: Perioperative chemotherapy for locally advanced gastric cancer in Japan: current and future perspectives

**DOI:** 10.1007/s00595-019-01950-2

**Published:** 2020-01-08

**Authors:** Masanori Tokunaga, Yuya Sato, Masatoshi Nakagawa, Tomoki Aburatani, Takatoshi Matsuyama, Yasuaki Nakajima, Yusuke Kinugasa

**Affiliations:** Department of Gastrointestinal Surgery, Tokyo Medical and Dental University, 1-5-45 Yushima, Bunkyo-ku, Tokyo, Japan

## Correction to: Surgery Today (2020) 50:30–37 10.1007/s00595-019-01896-5

The article Perioperative chemotherapy for locally advanced gastric cancer in Japan: current and future perspectives, written by Masanori Tokunaga, Yuya Sato, Masatoshi Nakagawa, Tomoki Aburatani, Takatoshi Matsuyama, Yasuaki Nakajima and Yusuke Kinugasa was originally published Online First without Open Access. After publication in volume 50, issue 1, page 30–37 the author decided to opt for Open Choice and to make the article an Open Access publication. Therefore, the copyright of the article has been changed to © The Author(s) 2020 and the article is forthwith distributed under the terms of the Creative Commons Attribution 4.0 International License (http://creativecommons.org/licenses/by/4.0/), which permits use, duplication, adaptation, distribution and reproduction in any medium or format, as long as you give appropriate credit to the original author(s) and the source, provide a link to the Creative Commons license, and indicate if changes were made.

The original article has been corrected.

